# Oxytocin evokes a pulsatile PGE2 release from ileum mucosa and is required for repair of intestinal epithelium after injury

**DOI:** 10.1038/srep11731

**Published:** 2015-07-10

**Authors:** Dawei Chen, Junhan Zhao, Haoyi Wang, Ning An, Yuping Zhou, Jiahui Fan, Junwen Luo, Wenlong Su, Chuanyong Liu, Jingxin Li

**Affiliations:** 1 Department of Physiology, School of Medicine, Shandong University, Jinan, 250012, People’s Republic of China

## Abstract

We measured the short-circuit current (*I*_sc_) in rat ileum mucosa to identify the effect of oxytocin (OT) on mucosal secretion in small intestine. We identified a COX-2-derived pulsatile PGE2 release triggered by OT in rat ileum mucosa. OT receptors (OTR) are expressed in intestine crypt epithelial cells. Notably, OT evoked a dynamic change of [Ca^2+^]_i_ in ileum crypts, which was responsible for this pulsatile release of PGE2. OT ameliorated 5-FU-, radiation- or DSS- induced injury *in vivo*, including the improvement of weight loss, reduced villus height and impaired survival of crypt transit-amplifying cells as well as crypt. Moreover, these protective effects of OT against intestinal injury were eliminated by coadministration of a selective inhibitor of PGE2, AH6809. Our findings strongly suggest that OT, a novel and important regulator of intestine mucosa barrier, is required for repair of intestinal epithelium after injury. Considering that OT is an FDA-approved drug, this work reveals a potential novel and safe way to combat or prevent chemo-radiotherapy induced intestine injury or to treat IBD.

The intestinal epithelium is the most vigorously self-renewing tissue of adult mammals. The four differentiated cell types that reside within the epithelium-goblet cells, enteroendocrine cells, paneth cells, and enterocytes. Proliferative cells reside in the crypts. The crypts harbor stem cells and their progeny, transit-amplifying cells (TACs). TACs spend approximately two days in the crypt, in which they divide 4–5 times before they terminally differentiate into the specialized intestinal epithelial cell types. Oxytocin (OT) is best known as a hormone produced in the paraventricular nucleus of the hypothalamus that stimulates OT receptors (OTR) to initiate milk ejection reflex and parturition^1^,^2^,^3^,^4^,^5^. OT/OTR, however, also participate in social behaviours, promoting trust and bonding^6^,^7^,^8^,^9^. In addition, OT has been shown to facilitate *in vitro* differentiation of mesenchymal stem cells towards cardiomyogenesis and osteogenesis^10^,^11^ and enhanced muscle stem cell activation/proliferation^12^. OT and OTR expression also has been demonstrated in bowel^13^,^14^,^15^. Enteric OT, like that of brain, is restricted to neurons; however, enteric OTRs are not exclusively neuronal. Enterocytes also express OTRs^14^. Intriguingly, OTRs is restricted to crypts, especially at crypt-villus boundaries^14^. Welch MG *et al*. recently demonstrated that OT/OTR signaling plays an important role in regulating the proliferation of crypt cells and mucosal permeability, and protecting from inflammation^16^.

Here, using short-circuit current (*I*_sc_) as dynamic biosensors of prostaglandins (PGs) release, we found that OT evokes a COX-2 dependent pulsatile release of prostaglandin E2(PGE2) by triggering [Ca^2+^]_i_ oscillations in ileum mucosa, and demonstrated that OT/PGE2 signalling pathway is required for intestinal epithelium regeneration. Importantly, we showed that systemic OT delivery alleviates chemo- and radiation-induced intestinal epithelium injury by improving TACs proliferation function and prolongs overall survival in mice, while pharmacologic attenuation of PGE2 signalling with a selective antagonist blocks the OT-induced protect effects. Our results suggest that this OT-evoked finely tuned release of PGE2 from intestinal epithelium could become a potential treatment to enhance host tolerance to aggressive chemoradiotherapy.

## Results

### OT evoked a continuous and pulsatile response in I_sc_ in rat ileum

**Figure 1:**
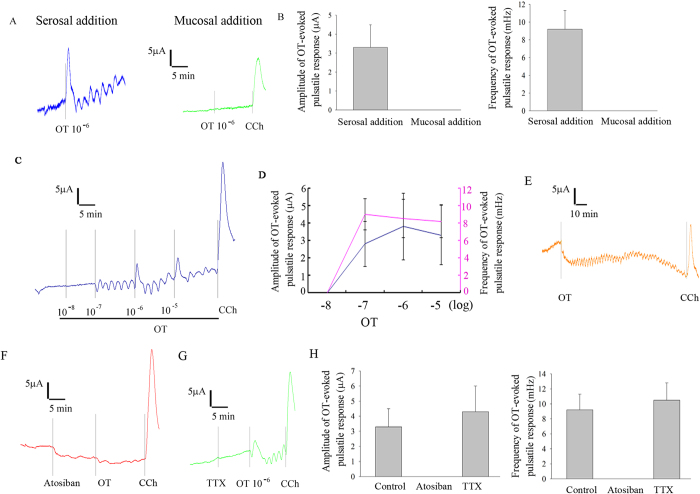
OT evoked a continuous and pulsatile response in *I*_sc_ in rat ileum. (**A**) Serosal but not mucosal administration of OT evoked a pulsatile response in *I*_sc_ in rat ileum, which has a 9.2 ± 2.1 mHz in frequency and 3.3 ± 1.2pA in amplititude (B, n = 7). The OT-evoked response was in dose-dependent manner (C-D, n = 4), which was sustained for at least 2 hours (Fig. 1E). Atosiban, but not TTX totally blocked the OT-evoked pulsatile response (F-H, n = 5).

The effect of OT on transepithelial ion transport was investigated using the Ussing chamber technique. The addition of OT (10^−6^ M) to serosal bathing solution evoked a pulsatile response in *I*_sc_ in rat ileum, however, the mucosal addition of OT did not affect basal electrical activity (Fig. 1A). The frequency and amplititude of OT-evoked pulsatile response was 9.2 ± 2.1 mHz, 3.3 ± 1.2 pA, respectively (Fig. 1B). This OT-evoked response is in a dose-dependent manner (Fig. 1C,D), which was sustained for at least 2 hours (Fig. 1E). To further investigate the underlying mechanism of this OT-induced *I*_sc_ response in rat ileum, atosiban, a specific blocker of OTR, was added to the bathing solution 15 min before the application of OT. Atosiban totally blocked the OT-evoked pulsatile response in *I*_sc_ (Fig. 1F). To investigate the involvement of the enteric nervous system (ENS) in the serosal OT-induced *I*_sc_ response. TTX (10^−6^M), a Na^+^ channel blocker, was added to the serosal bathing solution 15 min before the addition of OT. Unexpectedly, TTX did not affect the OT-evoked pulsatile response in *I*_sc_ (Fig. 1G,H).

### COX-2 dependent PGE2 release is responsible for the OT-evoked pulsatile response In I_sc_

**Figure 2:**
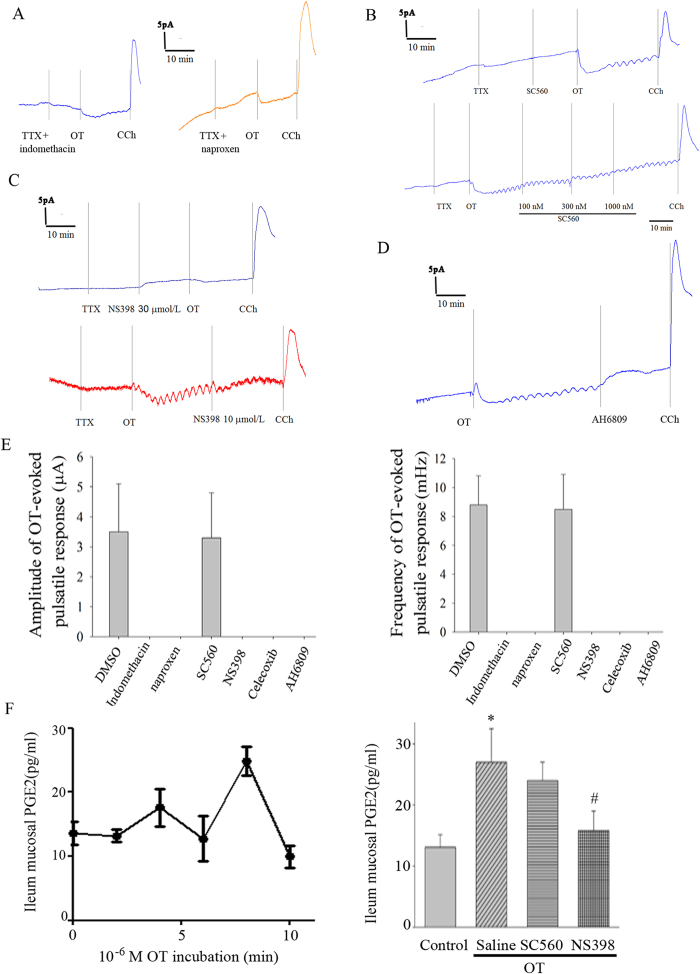
COX-2-derived PGE2 is responsible for the OT-evoked pulsatile response In *I*_sc_. Non-selective COX inhibitor indomethacin or naproxen (A and E) completely blocked the OT-evoked pulsatile response In *I*_sc_ in rat ileum (n = 6). The selective COX-2 inhibitor NS398 (C and E), but not COX-1 inhibitor SC560(B and E) totally blocked the OT-stimulated pulsatile response (n = 6). OT evoked a pulsatile PGE2 release in cultured rat ileum mucosa, which can be blocked by NS398, but not SC560 (F, n = 6). The EP2 antagonist, AH6809, completely blocked the OT-evoked pulsatile response (D,n = 5). *P < 0.05 versus control; #*P* < 0.05 *versus* Saline.

Next, we investigated the effect of a non-selective cyclooxygenase (COX) inhibitors (indomethacin and naproxen) in OT-evoked pulsatile response In *I*_sc_. The results showed that either non-selective COX inhibitor indomethacin or naproxen (Fig. 2A,E) completely blocked the OT-evoked pulsatile response in *I*_sc_ in rat ileum. To further determine the role of COX-1 or COX-2 in OT-evoked pulsatile response, a selective COX-1 inhibitor (SC560) or selective COX-2 inhibitors (NS398 and Celecoxib) were used. The selective COX-2 inhibitor NS398 (Fig. 2C,E) or Celecoxib totally blocked the OT-stimulated pulsatile response, but the selective COX-1 inhibitor SC560 (Fig. 2B,E) did not. ELISA results demonstrated that in the presence of OT, PGE2 was release from cultured rat ileum mucosa (Fig. 2F, left lane), which can be blocked by NS398, but not SC560 (Fig. 2F, right lane). To further confirm PGE2 involves in OT-evoked pulsatile response in rat ileum mucosa, the EP2 antagonist, AH6809 was used. Unsurprisingly, it completely blocked the OT-evoked pulsatile response (Fig. 2D-E). Moreover, the solvent DMSO has no effect on OT-stimulated pulsatile response (Fig. 2E). These findings suggest that OT-evoked pulsatile response in *I*_sc_ is mediated through PGE2 produced by COX-2.

### [Ca^2+^]_i_ oscillations in ileum crypt cells involve OT-evoked pulsatile PGE2 release

**Figure 3:**
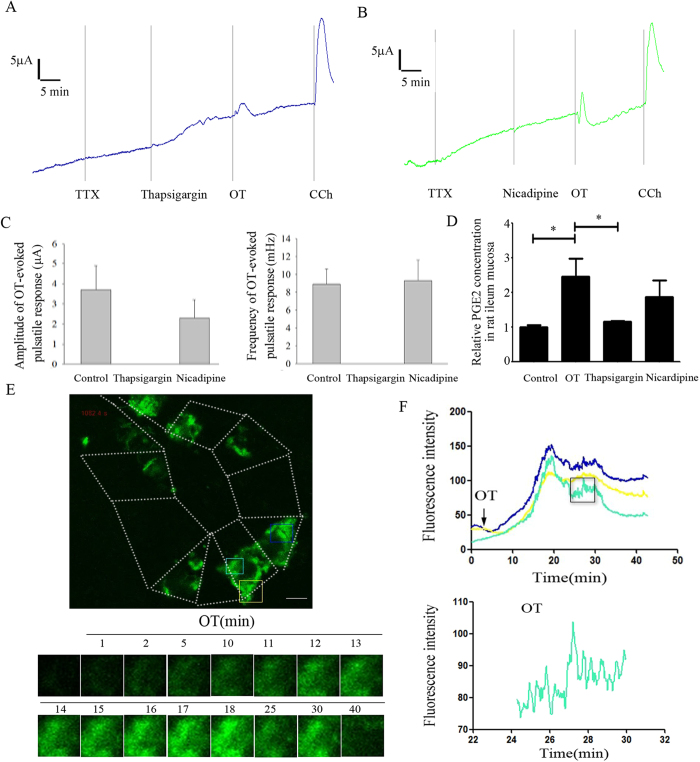
[Ca^2+^]_i_ oscillations in ileum crypt cells involve OT-evoked pulsatile PGE2 release. Thapsigargin, an intracellular Ca^2+^ pump blocker, but not nicadipine, a L-type Ca^2+^ channel blocker completely blocked the OT-evoked pulsatile response in *I*_sc_ (3A-C). Moreover, OT-induced PGE2 release was also largely inhibited by thapsigargin, but not nicadipine (3D, n = 5). Confocal Ca^2+^ imaging revealed that OT induces a dynamic change in cytosolic Ca^2+^ of crypt epithelial cells (3E-F). Scale bar: 10 μm. *P < 0.05.

To investigate whether Ca^2+^ involve in OT-evoked pulsatile PGE2 release, first, thapsigargin or nicadipine was used. Depletion of intracellular Ca^2+^ stores with thapsigargin eliminatedthe OT-evoked pulsatile response in *I*_sc_ but nicadipine, a L-type Ca^2+^ channel blocker, did not produce any effect (Fig. 3A–C). Moreover, ELISA results further demonstrated that OT-induced PGE2 release was largely inhibited by thapsigargin, but not nicadipine (Fig. 3D). Second, we tested the effect of OT on the [Ca^2+^]_i_ dynamics of epithelial cells in mouse ileal crypts. The spatiotemporal dynamics of [Ca^2+^]_i_ were measured by confocal imaging analysis in isolated crypts of mouse. Cells were loaded with the Ca^2+^ indicator dye fluo-3. Thereafter, OT induces an increase in cytosolic Ca^2+^ of crypt epithelial cells (Fig. 3E). Interestingly, OT-induced [Ca^2+^]_i_ dynamics of crypt epithelial cells have a spontaneous fluctuation pattern (Fig. 3F,G, Supplemental video). Taken together, these results hint that [Ca^2+^]_i_ oscillations of crypt epithelial cells involve OT-evoked pulsatile PGE2 release.

### OT protects intestinal mucosa by triggering the release of PGE2 from chemoradiotherapy-induced intestinal injury

**Figure 4:**
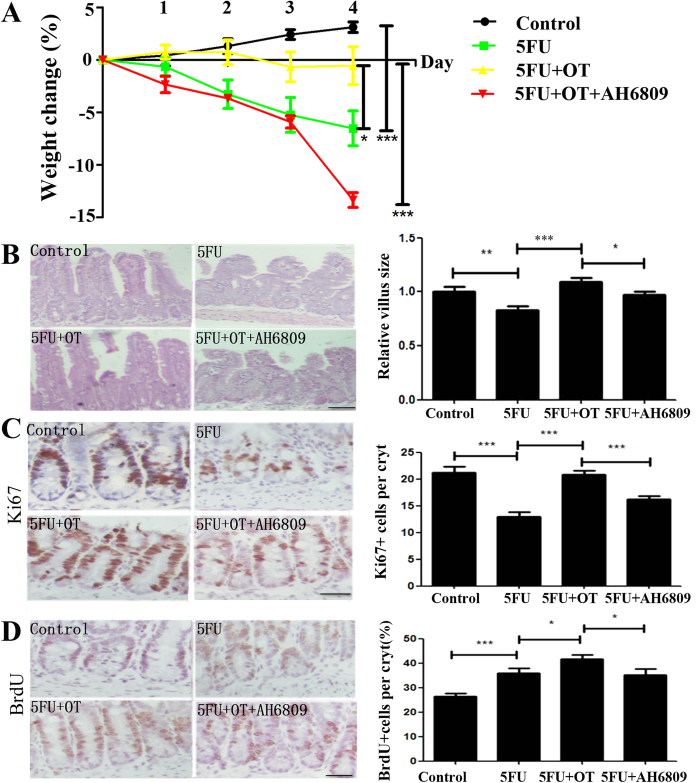
OT protects intestinal mucosa through PGE2 in 5-FU induced intestinal injury model. (**A**) The clinical severity of 5-FU-induced injury was tracked with relative weight loss as a surrogate marker (**B**) Effects of exogenous OT on 5-FU-induced intestinal morphology. Ileums from mice were stained with H&E, and the relative villus sizes were measured and statistically analyzed. Scale bar: 100 μm (**C**) OT protects the number and distribution of intestinal TACs after administration of 5-FU. Ileum from mice were immunohistochemically stained for Ki67^+^ cells. The numbers of positive cells were counted in each crypt. Scale bar: 50 μm (**D**) BrdU^+^ cells percent in each crypt represents the proliferation of intestinal stem cells. Scale bar: 50 μm. Results represent 30 tissue specimens in each group (n = 5) and the mean ± s.d. of six tissue sections per mouse. *P < 0.05; **P < 0.01; ***P < 0.001.

To investigate whether OT protects intestinal epithelium from chemoradiotherapy-induced intestinal injury. First, we used 5-FU injury model as chemotherapeutic model (30 mg/kg/d). After 5-FU treatment, body weight, ileum villus height and TACs number were significantly deceased (Fig. 4A-C). Administration of OT largely ameliorated 5-FU-induced injury either in weight loss and villus height, or TACs number. Moreover, AH 6809, behaved as an EP2 receptor antagonist, totally inhibited OT-induced protection in 5-FU-induced injury. In order to determine whether OT improves stem cells’s proliferative ability, we utilized BrdU as label. OT significantly increased the number of BrdU^+^ cells than in 5-FU-treated only group. Furthermore, AH6809 blocked OT-induced protection (Fig. 4D).

**Figure 5:**
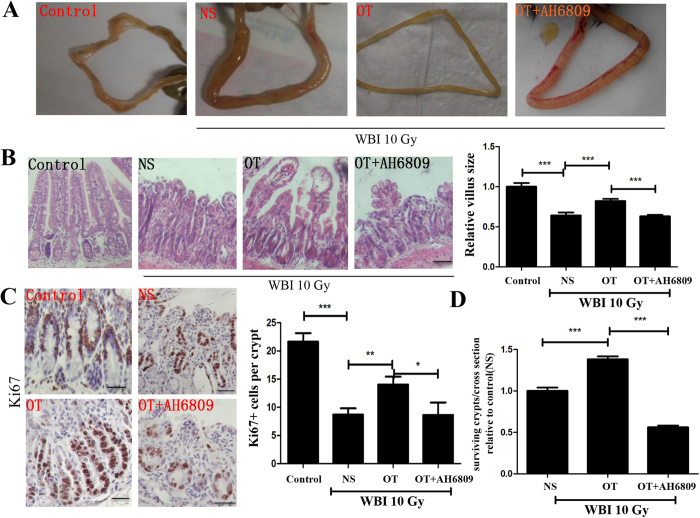
OT is radioprotective in small intestine epithelium. (**A**) Mouse was euthanized and ileum was pulled out to show the severity of intestinal edema. (**B**) Effects of exogenous OT on irradiated intestinal morphology. Ileum from mice were stained with H&E, and the relative villus sizes were measured and statistically analyzed. Scale bar: 100 μm (**C**) OT protects the number and distribution of intestinal TACs after irradiation Ileum from mice were immunohistochemically stained for Ki67^+^ cells. The numbers of positive cells were counted in each crypt. Scale bar: 50 μm (**D**) Crypt survival after irradiation in mice. Mice received 10 Gy of total body irradiation and were killed 3.5 days later. All animals received 5-bromo-2-deoxyuridine 2 h before death to label replicating epithelial cells. Results represent 30 tissue specimens in each group (n = 5) and the mean ± s.d. of six tissue sections per mouse. *P < 0.05; **P < 0.01; ***P < 0.001.

Second, we determine if OT is radioprotective in the intestine. Ten Gy whole body irradiation (WBI) exposure caused edema in the small intestine (Fig. 5A). Moreover, the villus height, the number of TACs or surviving crypts per intestinal cross section were also decreased after radiation (Fig. 5B–D). Administration of OT (0.1 mg/kg) 24 hours before radiation reduced the edema of the small intestine and increased either the villus height or the number of TACs (Fig. 5A–C). Notably, OT treatment increased the number of surviving crypts to 1.5-fold (Fig. 5D). To implicate PGE2 as a mediator of OT-induced radioprotection, EP2 antagonist AH6809 was used to determine if the radioprotective effects of OT were mediated through the induction of PGE2 synthesis. When AH6809 was administered with OT, the protective effects of OT in small intestine after radiation were lost (Fig. 5A–D).

Together, these findings suggest that the effects of OT as a novel chemo- and radio-protective agent are mediated through PGE2.

### OT alleviates dextran sulfate sodium (DSS)-induced intestinal injury

**Figure 6:**
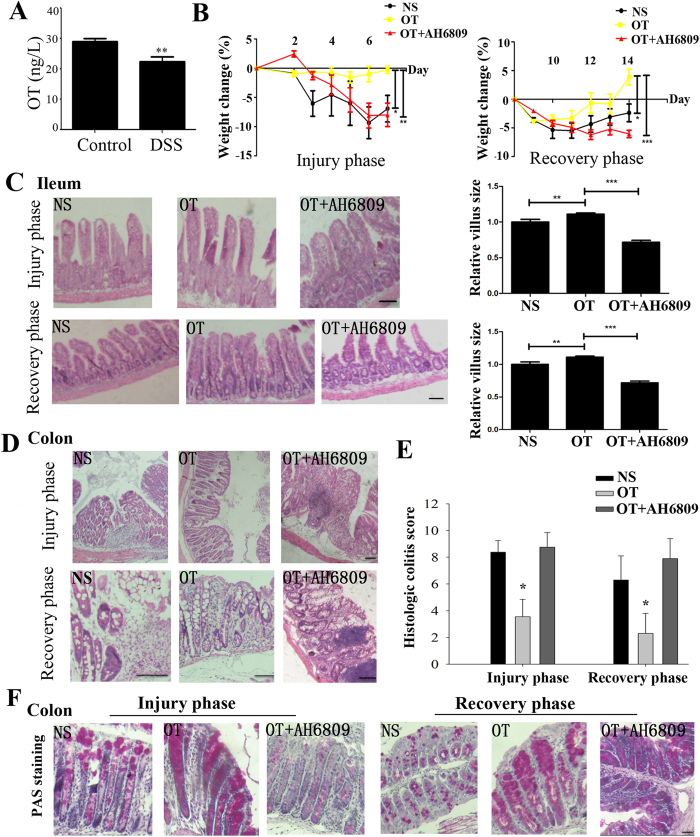
OT alleviates intestinal inflammation induced by DSS Mice were adminstered of 2.5% DSS for 7 days as injury phase, and the other groups of mice were treated with 2.5% DSS for 7 days followed by 7 days of recovery. (**A**) The concentration of OT was largely decreased in colon from DSS-induced mouse model (n = 5). (**B**) Mice weights were recorded each day and relative weight changed were regarded as measurement of injury degree (n = 5) (**C**) Ileum from mice were stained with H&E, and the relative villus sizes were measured and statistically analysed. Scale bar: 100 um (**D**) Representative images of mice colon H&E staining. Scale bar: 50 μm (**E**) Clinical colitis scores on the basis of histological sections of mice colon (**F**) PAS staining of colonic sections and the staining pattern on behalf of goblet cells secretion mucus. Scale bar: 50 μm. Results represent 30 tissue specimens in each group (n = 5) and the mean ± s.d. of six tissue sections per mouse. *P < 0.05; **P < 0.01; ***P < 0.001.

The murine DSS colitis model has been widely adopted to induce severe acute, chronic or semi-chronic colitis. After DSS, the concentration of OT was significantly decreased in colon from DSS-induced colitis mouse model (Fig. 6A) and the weight loss of OT treatment mice was significantly less than that of control mice in injury phase, and the gain weight of OT treatment mice was larger than that of control mice during the recovery period (Fig. 6B), which was concomitant with OT treatment mice displaying increased villus size (Fig. 6C) and elevated histological colitis scores (Fig. 6D–E). The colons from OT treatment mice exhibited a significant decrease in injury compared with control colons following DSS treatment characterized by epithelial cell loss, crypt damage, and immune cell infiltration (Fig. 6D).

In addition, a large increase in mucus production were observed in OT treatment mice by PAS staining (Fig. 6F).

## Discussion

OT is a mammalian neurohypophysial hormone, which acts primarily as a neuromodulator in the brain. In this study we show that OT evokes a pulsatile release of PGE2 in mouse ileum mucosal epithelium. Importantly, OT induced Ca^2+^ oscillations in ileum crypt is responsible for the pulsatile PGE2 release. Moreover, we found that OT is require for crypt cells proliferation, and is chemo-and radio-protective in the intestine and that its protective effects are mediated through the induction of PGE2 synthesis.

### A pulsatile PGE2 release from ileum mucosal epithelium evoked by OT is COX-2- and [Ca^2+^]_i_- dependent

PGs are known to play a role in ion transport across gastrointestinal epithelia. PGE2 increases chloride secretion in guinea pig colon^17^ and porcine small intestine^18^. In our study, chloride secretion was measured as *I*_sc_ utilizing Ussing chamber methodology. Therefore, the change of *I*_sc_ can be as dynamic biosensors of PGs release from mouse ileum mucosa. The key finding of the present work is that OT evokes a release of PGE2 from ileum mucosa. PGs are synthesized through two COX isoforms, COX-1 and COX-2^19^. COX-1 is a protein that is constitutively expressed in almost all tissues whereas the expression of COX-2 is highly regulated^20^. Several groups have reported that COX-2 also constitutively expressed in numerous tissues, including the kidney^21^, colon^22^, central nervous system^23^ and vascular endothelium^24^. Through the actions of either COX-1 or COX-2 (or both), arachidonic acid is converted to PGH2, which is then rapidly metabolized by various PG synthases to form PGs such as PGE2^25^. PGE2, the most abundant gastrointestinal PG, regulates many of the normal homeostatic functions of the gut, including motility and secretion^26^,^27^,^28^,^29^,^30^. Here we show that OT-evoked PGE2 release in mouse ileum mucosa is COX-2 dependent. Interestingly, OT-evoked release of PGE2 from ileum mucosa is oscillatory. Previous studies have demonstrated that OT induces [Ca^2+^]_i_ oscillations in human myometrial cells^31^ and in rat live cell line (Clone 9)^32^. Interestingly, they found the OT-induced [Ca^2+^]_i_ oscillations frequency was around 10 mHz, similarly to what we found in the present study. In mouse ileal crypts, some neurotransmitters can induce [Ca^2+^]_i_ dynamics of epithelial cells^33^. Furthermore, cytosolic calcium oscillations regulate a pulsatile PG release^34^.

Our present data demonstrated that OT stimulates relatively low frequency but large- amplititude [Ca^2+^]_i_ oscillations (i.e., ranging from approximately one oscillation every 10 min) in mouse ileum crypt cells, in which contains a relatively high frequency but small- amplititude oscillations (i.e., ranging from approximately one oscillation every 1 min). The large wave may be attributable to global Ca^2+^ feedback loops and the balance between Ca^2+^ release/entry and Ca^2+^ pumps, and the small Ca^2+^ oscillations presumably result from activation of PI-PLC via G protein-linked receptors, which results in sustained generation of IP_3_ and subsequent release of Ca^2+^ from IP_3_-sensitive stores in the endoplasmic reticulum balanced by the actions of Ca^2+^ pumps that lower intracellular Ca^2+^.

**Figure 7:**
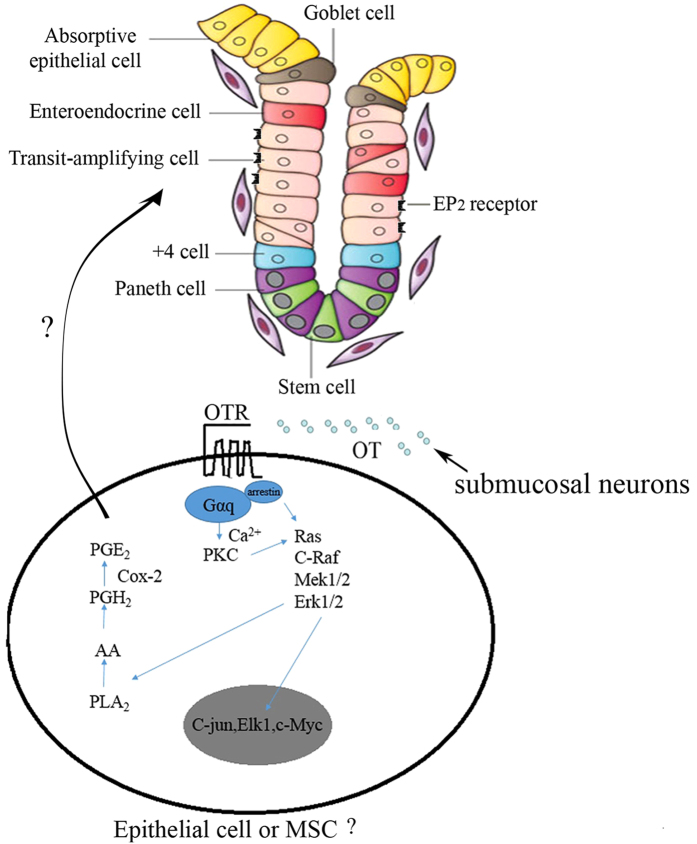
Proposed mechanisms for OT-evoked PGE2 release in rat ileum mucosa. OT, released from enteric neuron, activates PKC and ERK upon binding with its receptors in intestine epithelial cells or other cells of the lamina propria, induces the increase of [Ca^2+^], then triggers the expression of COX-2 and thus evokes a PGE2 release.

Beside ENS, ileum crypt cells, especially at crypt-villus boundaries also expressed OTR^14^. It may be a direct effect of OT on epithelial cells. In the myometrium, OT binds to a specific G protein-coupled receptor (GPCR) linked through Gα_q/11_ to phospholipase Cβ (PLCβ). Activation of PLCβ results in the production of inositol 1, 4, 5-trisphosphate (IP_3_) and diacylglycerol (DAG)^2^. IP_3_ releases Ca^2+^ from intracellular stores. OT induces COX-2 expression mainly by activating PKC and ERK in human myometrial cells^35^. Intracellular Ca^2+^ triggers the expression of COX-2 by activating PKC-α and calmodulin kinase II (CaMKII) cascades^36^. Therefore, we hypothesis that OT, released from enteric neuron, evokes a PGE2 release via OTR/PKC or ERK/Ca^2+^ in ileum cryt cells (Fig. 7).

### OT involves in the repair after intestine epithelial injury

PGE2 has been reported to be important for the regulation of stem cells growth and differentiation^37^,^38^,^39^. Furthermore, PGE2 increases intestinal crypt stem cells survival^40^,^41^. Previous research have shown OTR expression was restricted in crypts and concentrated on crypt-villus junction^14^, and TACs occupied this position primarily^42^. The crypt is a proliferative compartment which is composed of 250–300 cells that are in constant active proliferation; it also generates all of the cells required to renew the entire intestinal epithelium in mice in 2–3 days. Recent research reveals that Villi and crypts were shorter in OTR knockout mice than in wild-type mice and TAC proliferation in OTR knockout mice crypts was deficient^16^. We also confirmed that OTR expressed in TACs, and OT increased the crypt cell proliferation (Supplemental figure1). Using chemo- or radio-therapy-induced mouse models of intestinal epithelial injury, we further confirmed that the OT is protective by promoting the crypt cells proliferation. Exogenous or endogenous PGE2 have been shown to be radioprotective^40^,^43^,^44^ in the intestine. There are 4 known receptors for PGE2 (EP1, EP2, EP3, and EP4). Two of these (EP2 and EP4) are expressed in the crypt cells from human colon and inflamed small intestine^45^. Our present study shows that treatment with OT increased total intestinal PGE2 levels 2.5-fold, and AH6809, an EP1/2 antagonist, blocked the OT-evoked protection against chemo- and radio-induced injury.

Three pieces of indirect evidence also suggest that PGE2 is the mediator. The first is that PGE2 is radioprotective in the intestine^44^ and AH6809 worsens the chemo- and radio-induced injury; the second is that PGE2 is the most abundant prostaglandin in the intestinal epithelium^46^; and the third is that PGE2 is known to modulate stem cell survival^41^.

PGs are degraded very rapidly and usually act on cells near the cells in which they are produced. The simpler and more likely explanation is that PGE2 produced by crypt epithelial cells acts on stem cells to induce protection.

This suggests that OT-induced protection may be mediated by PGE2 produced by crypt epithelial cells themselves.

Inflammatory bowel disease (IBD) is a group of chronic inflammatory conditions with unknown pathogenesis. IBD comprised of Crohn’s disease (CD) and ulcerative colitis (UC), are chronic diseases characterized by aberrant immune responses to luminal bacteria in genetically susceptible subjects^47^. The colonic epithelium is covered by a continuous mucus layer, which serves as an important barrier and prevents colonic bacteria from invading the mucosa and cause inflammation. The mucus layer also functions as a shelter that is required to retain a bacterial colonization within the colon and prevents the bacteria from constantly being removed by peristalsis. Thus colonic mucus has dual functions, being a functional barrier to avoid contact of bacteria to the epithelium and simultaneously enabling bacteria persistence within the colon. There is a correlation between decreasing mucus barrier and increasing clinical symptoms during onset of colitis^48^. In our present study, we found OT alleviated DSS-induced colitis. Interestingly, OT also increased the mucus production in colon.

In conclusion, our results reveal that OT evoked a pulsatile PGE2 release from intestine mucosa, and is responsible for the repair after intestine epithelial injury. There may be therapeutic potential for OT in protecting the intestinal epithelium in patients receiving chemotherapy or radiation therapy. Considering that OT is an FDA-approved drug, this work reveals a potential novel and safe way to combat or prevent chemo-radiotherapy induced intestine injury or to treat IBD.

## Methods

### Animal models

C57BL6/J mice and Wistar rats were purchased from the Animal Centre, Shandong University, China. 6–8 weeks male mice were divided with randomization. In crypt stem cell proliferation experiment, mice were intraperitoneally injected with normal saline (NS) or OT (1 mg/kg/day) for 14 days. In 5-fluorouracil (5-FU)-induced intestinal injury model, mice were given 5-FU (30 mg/kg/day for 5 days for the therapeutic dose). A radiation-induced intestinal injury model was established in mice by whole body irradiation (10 Gy total body irradiation using a 6MV-X linear accelerator). For DSS-inducedinjurymodel, 2.5% DSS salt (reagent-grade, mol. wt. 36 to 50 kDa; MP Biomedicals) in autoclaved drinking water was given to mice for 7 days as injury phase, for recovery studies, DSS was administered for the first 7 days as indicated, and then DSS was removed from the drinking water and mice were killed 7 days after the cessation of DSS treatment. One day before chemical treatment or 10 Gy irradiation, Mice were injected intraperitoneally normal NS, OT (0.1 mg/kg/day) or OT +AH6809(0.5 mg/kg/day). Animals were killed after chemical treatment or irradiation, and rapidly dissected, as described previously^41^. The proximal jejunum was fixed before paraffin embedding and immunohistochemical analysis. Crypt survival was counted in animals killed 3.5d after irradiation using a modification of the microcolony assay^49^. 2 hours before death, 5-bromodeoxyuridine (BrdU)(120 mg/kg) and 5FU (12 mg/kg) was injected to each mouse to label S phase cells. The 5 μm paraffin sections were prepared from ileum oriented so that the sections were cut perpendicular to the long axis of the small intestine. The viability of each surviving crypt was confirmed by immunohistochemical detection of BrdU incorporation into five or more epithelial cells within each regenerative crypt in at least six intergtal sections. Histologic evaluation was performed by two investigators blinded to the animal groups and the inflammation was graded as follows: severity of inflammation (0–3: none, slight, moderate, severe), extent of injury (0–3: none, mucosal, mucosal and submucosal, transmural), and crypt damage (0–4: none, basal 1/3 damaged, basal 2/3 damaged, only surface epithelium intact, entire crypt and epithelium lost).

All experimental procedures were conducted in accordance with the Guidelines for the Care and Use of Laboratory Animals of Shandong University, and the study was approved by the Medical Ethics Committee for Experimental Animals, Shandong University, China (number ECAESDUSM 2012029).

### *I*_sc_ measurement

Rats, weighting between 200 and 250 g, were used for this study. Animals were fasted overnight with free access to water before experiments, and then anesthetized and decapitated. Segments of ileum were cut along the mesenteric border, and luminal contents were gently removed. Tissues were pinned flat on a Sylgard-lined Petri dish with mucosal surface down, and tissue preparations were constructed by using forceps and scissors. To obtain the mucosal-submucosal preparations, the serosa and muscularis were gently stripped away. During preparation, tissues were bathed in ice-cold Krebs solution (bathing solution) and continuously oxygenated with a gas mixture of 95%O2 and 5%CO2. Krebs solution contained (in mM) 120.6 NaCl, 5.9 KCl, 2.5 CaCl2,1.2 KH2PO4, 1.2 MgCl2, 15.4 NaHCO3 and 11.5 glucose.

*I*_sc_ was measured *in vitro* in Ussing chamber technique. The tissue preparations were mounted between the 2 halves of the Ussing chambers (exposed area of 0.50 cm^2^), equipped with water-jacketed gas lifts, and were bathed on both sides with 5 mL Kerbs solution, gassed with 95% O_2_ and 5% CO_2_, pH adjusted to 7.4, and maintained at 37 °C by circulating the solution through a reservoir during the experiments. The tissue was continuously voltage-clamped to zero potential difference by the application of external current, with compensation for fluid resistance. The baseline value of the electrical parameters was determined as the mean over the 3 min immediately prior to drug administration. The tissues were allowed to rest for approximately 10 min to stabilize the *I*_sc_ prior to the addition of drugs. The transepithelial potential difference for each preparation was measured with Ag/AgCl reference electrodes (P2020S; Physiologic Instruments, San Diego, Calif) connected to a preamplifier that was, in turn, connected to a voltage clamp amplifier (VCC MC4; Physiologic Instruments, San Diego, Calif). To check tissue availability, tissues were stimulated by carbachol (CCh). Changes in *I*_sc_ were measured. Each drug was dissolved in distilled water or DMSO solution for stock solution and added to the bath to provide the desired molar concentration. Thapsigargin (1 μM) and nicadipine (1 μM) were used to investigate whether Ca^2+^ involved in OT-evoked *I*_sc_ changes.

### Immunofluorescent and immunohistochemistry

The ileum was open longitudinally and flushed by cold PBS until most of the luminal content were clear. The tissues were fixed in 4% paraformaldehyde overnight and embeded in paraffin wax. Then the tissues were sectioned (3–4 μm slices). After dewaxing and hydration, antigen retrieval was performed by boiling slices for 30 min in 10mM sodium citrate buffer pH6.0. Samples were blocked with 3% BSA (IHC grade) and incubated with primary antibody at 4 °C overnight, Antibodies used were rabbit anti-ki67(1:200, cell signaling), mouse anti-BrdU (1:400, cell signaling), goat anti-oxytocin receptor (1:100, Abcam) rabbit anti-lysozyme (1:200, DAKO). For immunohistochemistry, the VECTASTAIN Universal Quick Kit, R.T.U. (Ready-to-Use) kit (vectorlabs) was used as secondary regents. Stainings were developed with DAB. Slides were counterstained with haematoxylin and mounted for observation under a Nikon eclipse 80i light microscope. The immunohitochemistrical staining data were analysed double blindly using image-pro plus software. For immunofluorescent, Alexa Fluor® 568 donkey anti- rabbit and Alexa Fluor® 488 donkey anti-goat (life technologies) were used as secondary antibodies. Then sections were counterstained with DAPI. All slides were imaged with a Zeiss LSM780 laser scaning confocal microscope.

### The measurement of PGE2 and OT

The small intestinal tissues were mounted between the 2 halves of the Ussing chambers (exposed area of 0.50 cm^2^), equipped with water-jacketed gas lifts, and were bathed on both sides with 5 mL Kerbs solution, gassed with 95% O2 and 5% CO2, pH adjusted to 7.4, and maintained at 37 °C by circulating the solution through a reservoir during the experiments. For time test experiment, OT (1 μM) was given 0,2,4,6,8,10 min before tissues were homogenated and centifuged for ELISA at 4 °C. For measurement of PGE2 in response to nicardipine and thapsigargin,10 min before OT treatment, DMSO, nicardipine or thapsigargin was added, respectively, then the tissues were collected for PGE2 ELISA (R&D). For OT measurement, the mouse distal colon were cut and homogenized. After two freeze-thaw cycles, the homogenates were centrifuged for 5 minutes at 5000 rcf, 4 °C. The supernatant was removed and assayed immediately by a mouse OT elisa kit (Mybiosource)‍. **Crypt isolation and confocal calcium imaging**.

Crypts were isolated as previously described^50^. Specifically, mouse small intestine (about 25 cm) were dissected out of the animals flushed with ice-cold sterile PBS, cut open lengthwise and into 1 cm pieces, and transferred into the Petri dish with ice-cold sterile PBS supplemented with 1% penicillin/streptomycin (Life Technologies). Tissue fragments were incubated with 2 mM EDTA in PBS for 30 min on ice. After removal of EDTA, tissue fragments were washed with PBS to release crypts. The tube was shaken by hand for 3 to 5 minutes. The villi and mucus were removed with a 70-mm cell strainer (BD Biosciences). After being centrifuged at 4 °C at 300 rcf for 5 minutes, the crypts was washed once with 1x PBS, and treated with TrypLE Express (Life Technologies) for 30 min at 37 °C to dissociate cell-to-cell attachment. Then, the dissociated cells were passed through a 40-μm strainer and subsequently, through a 20-μm strainer. The strained cells were pelleted, resuspended in PBS + 2% FBS, and used as single crypt cells. Then the crypts were incubated with 2 uM fluo-3, AM for 30 min at 37 °C, washed by Kerbs solution and observed under a Zeiss LSM780 laser scaning confocal microscope after 1 μM OT treatment.‍

### Statistical analysis

The experimental data were statistically analyzed by two-tailed Student’s *t*-test and one-way ANOVA, respectively,to compare single and multiple means. A *P* value of less than 0.05 or 0.01 was considered statistically significant or very significant, respectively.

## Additional Information

**How to cite this article**: Chen, D. *et al*. Oxytocin evokes a pulsatile PGE2 release from ileum mucosa and is required for repair of intestinal epithelium after injury. *Sci. Rep*. **5**, 11731; doi: 10.1038/srep11731 (2015).

## Supplementary Material

Supplementary Information

Supplementary Video
