# Supplementary material for: Oxytocin evokes a pulsatile PGE2 release from ileum mucosa and is required for repair of intestinal epithelium after injury
Source: Sci Rep. 2015 Jul 10;5:11731. doi: 10.1038/srep11731 (PMC4498177; doi:10.1038/srep11731)
Supplement: Supplementary Information [file srep11731-s1.pdf]

Oxytocin evokes a pulsatile PGE2 release from ileum mucosa and is required for repair of intestinal epithelium after injury

Dawei Chen*, Junhan Zhao*, Haoyi Wang, Ning An, Yuping Zhou, Jiahui Fan, Junwen Luo , Wenlong Su, Chuanyong Liu and Jingxin Li [#]

1. Department of Physiology, School of Medicine, Shandong University, Jinan, 250012, People's Republic of China

* These authors contributed equally to this work.

#Corresponding author. Tel: +86-0531-88382395; fax: +86-0531-88382502;

Email: ljingxin@sdu.edu.cn;

postal address: Department of Physiology, Shandong University School of Medicine. Jinan 250012, China

Supplemental figure 1

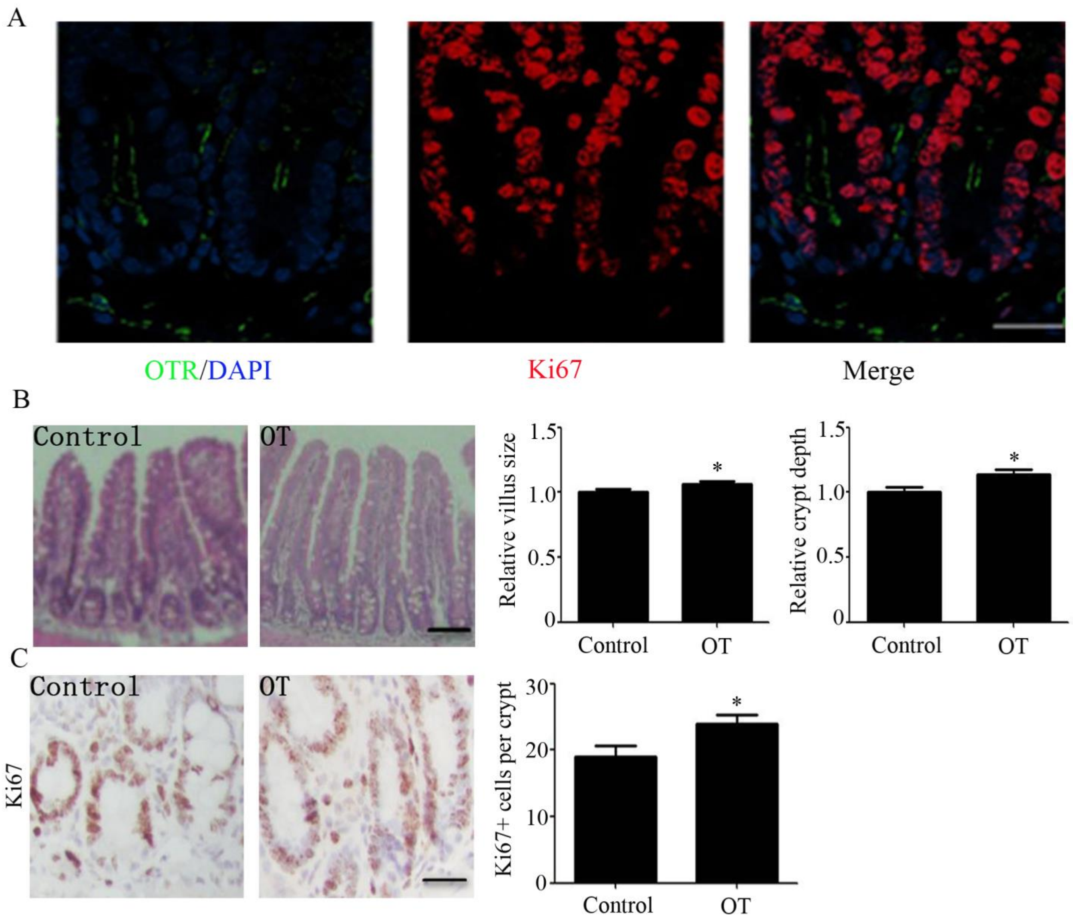

OTR expressed in TACs, and OT influence the proliferation of small intestine crypt cells

(A) Mouse ileum stained for OTR(green),Ki67(red) and DAPI (blue) shows Ki67$^+$ cells expressed OTR. Scale bar: 50 μm

(B) Effects of exogenous OT on intestinal morphology. Ileums from mice were stained with H&E, and the relative villus sizes and crypt depth were measured and statistically analyzed. Scale bar:100 μm

(C) OT affects the number and distribution of intestinal TACs. Ileums from mice were immunohistochemically stained for Ki67$^+$ cells. The numbers of positive cells were counted in each crypt. Scale bar:50 μm.

Results represent 30 tissue specimens in each group (five mice per group) and the mean ± s.d. of six tissue sections per mouse. *, P<0.05.

Video abstract

The small intestinal crypts were isolated. After 4 hours of culture, the crypts were incubated with 2 μM fluo-3, AM for 30 min at 37 ℃ ,washed by Kerbs solution and observed under a Zeiss LSM780 laser scaning confocal microscope. Confocal calcium imaging reveals OT-evoked intracellular Ca^{2+} elevation and oscillations in ileum crypt cells.
